# Identification of appropriate tools to gauge brain functions in a clinical setup of a developing country: A pilot study

**DOI:** 10.12669/pjms.39.6.7489

**Published:** 2023

**Authors:** Umema Zafar, Syed Hamid Habib, Syed Shahmeer Raza

**Affiliations:** 1 Umema Zafar, MBBS, MPhil, CHPE Department of Physiology, Rehman Medical College, Peshawar, Pakistan; 2 Syed Hamid Habib, MBBS, PhD, PGD, DHPE, CHR, CRSM, CME Department of Physiology. Institute of Basic Medical Sciences, Khyber Medical University, Peshawar, Pakistan; 3 Syed Shahmeer Raza, MBBS, MPhil, CRSM, ATC Department of Physiology, Gajju Khan Medical College, Swabi, Pakistan

**Keywords:** Vision, Hearing, Cognition, Motor, Emotions, Tools, Scales

## Abstract

**Objective:**

To identify the most appropriate tools to measure functions of the brain that can be utilized in the clinical setups of developing countries.

**Methods:**

This qualitative research with a three-step approach was carried out from January 2022 to May 2022 at the Institute of Basic Medical Sciences, Khyber Medical University, Pakistan. Firstly, literature was searched to identify main brain faculties, then interviews were conducted with regional field experts to identify appropriate scales for the selected functions. Lastly a rubric was filled using interview transcripts and literature.

**Results:**

The identified functions were vision, hearing, cognition, motor and emotions. Based on the rubric the best tests were visual fields (17/24), pure tone audiometry (16/24), Mini-Mental State Exam (20/24), Trait Emotional Intelligence Questionnaire (18/24), Romberg’s test (19/24) and Manual Muscle Testing (18/24).

**Conclusion:**

The clinicians in developing countries can utilize the visual fields, pure tone audiometry, Mini-Mental State Exam, Trait Emotional Intelligence Questionnaire, Romberg’s test and Manual Muscle Testing for most efficient, feasible, accurate and cost-effective measurement of brain functions.

## INTRODUCTION

Brain is a complex organ that receives information via the sensory receptors, modifies it, and acts through the effectors, be it motor, endocrine, or generating new thoughts.^1^ The American Heart Association/American Stroke Association (AHA/ASA) defined brain health as; ”Average performance levels among all people at that age who are free of known brain or other organ system diseases in terms of decline from function levels, or as adequacy to perform all activities that the individual wishes to undertake”.^2^ Diverse tools can be used to quantify different faculties of brain corresponding to various brain areas. For example, motor function corresponding to the motor cortex, cerebellum, and basal ganglia can be assessed by hand dexterity, balance, and strength/power.^3^ Similarly, if a person has normal cognitive abilities like working memory and executive function, this indicates his prefrontal cortex is in good health.^4^ Three ways to gauge brain health as opposed to brain function are; to check the brain’s anatomical, physiological, and biochemical characteristics.^5^ The brain has been divided into 52 areas by Brodmann, each area having unique anatomy and function.^6^,^7^ These brain areas can be broadly placed in ten individual and overlapping categories as identified by Brodmann and later subcategorized by Ferng.^6^ Various tools can be used to measure brain functions some of these are listed below.

The Mini-Mental State Exam (MMSE) or the Weschler’s scale are widely used for cognitive functions.^8^,^9^ Hearing can be measured by Pure Tone Audiometry (PTA) or via Brain Evoked Response Audiometry (BERA).^10^ Traits like visual function and emotional well-being can be quantified using Visual acuity, visual fields for vision, and Trait Emotional Intelligence Questionnaire Short Form (TEIQue-SF) for emotional well-being.^11^ A properly executed motor actions require balance, strength, dexterity, endurance and visual-motor coordination to be apt. A motor action can have all or a combination of these.^12^ Out of these motor strength can be checked both subjectively and objectively and can be used as a good representative of motor function.^6^

There are limitations for using diagnostic tests in a tertiary care hospital in a developing country.^13^ For example, cost-effectiveness, the expertise of technicians/health staff, availability of infrastructure for providing an optimum environment for the instruments, and maintenance of the equipment. Another important limitation is the time of application of the test/scale, due to the increased load of the sick in the hospitals.^14^ Due to the same reason, there is a need to identify the most efficient, cost-effective, accurate, and easy-to-use scales for measuring brain functions in the developing world. This research aims to identify the most appropriate tools for measuring brain health in a tertiary care clinical setup in a developing country.

## METHODS

This qualitative research was conducted at Institute of Basic Medical Sciences, Khyber Medical University, Pakistan from January to May 2022. It was approved by the ethical board of KMU vide no. KMU/IBMS/IRBE/meeting/2022/8072, and consisted of a three-step approach. The first step involved identifying the functions of the brain from literature and narrowing them down to five. Literature search was carried out using keywords such as “tools for brain health”, “functions of the brain” and “tools for checking brain functions in a clinical setup”. The second step involved interviews with field experts using open-ended questions. This was carried out to explore the scales used to measure those six functions of the brain. In the third step, a rubric consisting of eight criteria was developed, and the rubric was filled using both interviews and literature search. After the interviews, the scales identified were searched in literature to fill the rubric (Table-I).

**Table-I:** Rubric with grading for choosing the appropriate Scales

Rubric for the Scales				

Criteria S.no.	Criteria	Poor indicator 1 point	Average indicator 2 points	Good indicator 3 points
Criterion 1	Price per test for patient (dollars)	More than 5 dollars	1-5 dollars	free
Criterion 2	Portability	Completely immobile to one place	Can be moved within an institute	Fully portable
Criterion 3	Level of skill needed for use	Proper qualification required	Training needed	No prior training
Criterion 4	Assessment time	More than 15 minutes	6-15 minutes	1-5 minutes
Criterion 5	AUC	0.6<AUC<0.8	0.8<AUC<0.9	0.9<AUC<1.0
Criterion 6	Diagnosing Accuracy/validity/ reliability	Less than 69%	70-89%	90-100%
Criterion 7	Usage in clinical setup/ research	Usage less than 49%	Usage between 50-79%	Usage more than 80%
Criterion 8	Link with brain health	Less than 5	6-8	9-10

The interviews were conducted with ophthalmologists, ENT specialists, Neurologists and Psycho-physiologists. Twelve experts having more than ten years of experience in their relevant specialty were selected. The interview lasted for about 10-20 minutes each. It was either done in person or using the zoom (online web hosting) platform. The interviews were recorded after taking consent from the interviewee/consultant. This was done using a cellular smartphone and field notes were also taken.

After each interview, data were transcribed verbatim and field notes were added to it. The audio-recording and transcripts were cross-checked by the Principal Investigator to improve the validity and reliability of the data analysis. The approach used was grounded theory, in which deductive analysis of the data was done.^15^

### Participant involvement:

Participants were not actively involved in the design and choice of outcomes. However, they were involved in the conduct of the research. The research interview questionnaire was devised keeping in view the expertise of the participants/field specialists. There is no provision for dissemination of the research results to the interviewees except via academia and publication.

## RESULTS

The five faculties identified in the literature search were; visual function, hearing, cognition, motor function, and emotional wellbeing. The range of scores obtained were from 12-20 (from the possible range of 8-24). The details of which are mentioned in Table-I.

Following deductions were extracted from the interviews with field experts:

The best test to gauge visual function is visual fields and of all the other tests it has the greatest link with brain function. The second in line is visual acuity which, has a lesser link, due to its ease of conductance and feasibility (Table-II). The best tool to measure hearing ability is pure tone audiometry in our setup. The tool having maximum link with optimum brain functioning is Brain Evoked Response Audiometry (BERA) (Supplementary Table-I). The best method to check handgrip strength is Manual Muscle Testing. The best test for balance is Romberg’s test others include tandem walking and the Unterberger test (Supplementary Table-II). For cognition, it is Mini-Mental State Examination (MMSE) as well as Weschler’s scale and for emotional well-being is Trait Emotional Intelligence Questionnaire (TEIQue-SF) as it is validated in the local language and is easy and quick to administer (Table-III).

**Table-II:** Scoring of tools according to the Rubric devised.

S No.	Brain functions	Tools		C1: Price per test for patient	C 2: Portability	C 3: Level of skill needed for use	C 4: Time for assessment	C 5: AUC	C 6: Diagnosing Accuracy	C 7: Usage in clinical setup	C 8: Link with brain health	Total
1.	Visual Function	Visual Acuity		2	1	2	1	3	3	3	2	17
2.		Visual fields		3	2	2	2	2	2	3	1	17
3.	Hearing function	Pure tone audiometry		2	2	2	2	1	3	3	1	16
4.		Brain evoked response audiometry		1	2	2	2	1	3	1	1	13
5.	Cognition	Wechsler’s scale		1	3	1	1	2	1	1	3	13
6.		MMSE scale		3	3	3	2	2	2	2	3	20
7.	Emotional well being	TEIQue-SF		3	3	3	2	2	2	1	2	18
8.		MSCEIT		1	3	1	1	2	1	1	2	12
9.	Motor function	Hand grip strength	Dynamometry	3	3	3	3	1	2	1	2	18
10.			Manual Muscle Testing	3	3	2	3	1	1	3	2	18
11.		Balance	Romberg’s test	3	3	3	3	1	1	3	2	19
12.			Unterberger’s test	3	3	3	3	1	1	2	2	18
13.			Tandem walking test	3	3	3	3	1	1	2	2	18

C1-C8: Criterion 1 -Criterion 8, MMSE: Mini-Mental State Examination, TEIQue-SF: Trait Emotional Intelligence Questionnaire Short Form, MSCEIT: Mayer–Salovey–Caruso Emotional Intelligence Test. Based on Table-II the following tools were selected to measure the brain's functions after using the rubric (Table-I):1. Visual fields, 2. Pure tone audiometry, 3. MMSE scale, 4. Manual Muscle testing, 5. Romberg's test, 6. TEIQue-SF.

**Supplementary Table-I:** Selective interview questions along with responses from ophthalmologists and ENT specialists.

Interview Questions (Briefed)	Field expert responses (verbatim)
***Visual function***	
Best technique for measuring visual function based on its feasibility, most common usage and ability to diagnose visual impairment?	Response 1, 2 &3: Visual fields
Reason to the previous answer	Response 1: Visual acuity is limited in the sense that it only measures functions of the fovea where maximum vision is. If visual field is reduced to 5 degrees temporally and nasally with 6/6 visual acuity, person’s visual function is grossly deranged despite having normal visual acuity.
Do you think optimum visual function has a link with brain health? If Yes Why?	Response 3: Vision both central and peripheral is a function represented in the occipital cortex with wide range of connections with other brain areas. Hence, when vision is affected then brain function is also compromised.
***Hearing function***	
Best technique for measuring hearing ability based on its feasibility, most commonly used and ability to diagnose hearing impairment?	Response 1&3: Evoked Response Audiometry (ERA) Response 2: Audiometry
Reason to the previous answer	Response 1&3: ERA is more sensitive test and is less subjective than audiometry. Response 2: Audiometry is less expensive more readily available in clinical setup and usually done in adults.
Do you think optimum hearing ability has a link with brain health? If Yes Why?	Response 1: Yes, if seen in terms of health of auditory cortex. For example damage to auditory pathway can cause it to atrophy and can lead to development of other brain areas as a compensation. Response 2: Hearing ability does not have much relation with brain health as in sensorineural deafness even in some forms of conductive deafness problem is mostly peripheral like in cochlear nerve.

**Supplementary Table-II:** Selective interview questions along with responses from Neurologists

Interview Questions (Briefed)	Field expert responses (verbatim)
***Hand grip strength***	
Best technique for measuring visual function based on its feasibility, most common usage and ability to diagnose reduced grip strength?	Response 2 &3: Manual Muscle Testing Response 1: Dynamometry
Reason to the previous answer	Response 2: Easily done, also takes lesser time Response 3: Feasible, easy to do and the scale response is recognized and understood worldwide. Response 1: It gives an objective assessment of hand grip strength in Kgs.
Do you think optimum hand grip strength has a link with brain health? If Yes Why?	Response 2: Yes, it gives information about the integrity of central nervous system.
***Balance***	
Best technique for measuring hearing ability based on its feasibility, most commonly used and ability to diagnose loss of balance/vertigo?	Response 1: Romberg’s test Response 2: Gait testing Response 3: Unterberger’s test
Reason to the previous answer	Response 1: It takes away the vision hence, excludes that cause and highlights the cerebellar cause. Response 2: It provides maximum information in minimum time.
Do you think optimum balance has a link with brain health? If Yes Why?	Response 2: Yes, all brain processes are linked for example by exercises like yoga that improve increase concentration and memory. Response 3: Balance is a product of receptors, muscles, vision, inner ear and cerebellar functioning. If balance is affected other brain functions or parts like cerebellum might also be affected.

**Table-III:** Selective interview questions along with responses from Psychophysiologists

Interview Questions (Briefed)	Field expert responses (verbatim)
***Emotional well being***	
Best technique for measuring emotional well being based on its feasibility, most commonly used and ability to diagnose emotional disturbance?	Response 1 & 2: Trait Emotional Intelligence Questionnaire Short Form Response 3: Mayer–Salovey–Caruso Emotional Intelligence Test
Reason to the previous answer	Response 2: Urdu version of TEIQue-SF is a reliable and valid measure to assess trait emotional intelligence in Pakistani adult population. It’s also quick and easy to administer as compared to other scales.
Do you think optimum emotional well-being has a link with brain health? If Yes, Why?	Response 3: Emotional well-being is how people manage and cope with their emotions. It has a direct link with brain and mental health. As it helps us to manage stress, develop social relations and care for ourselves. All these if done effectively in turn promote brain health.
***Cognitive function***	
The best technique for measuring cognition based on its feasibility, most commonly used and ability to diagnose cognitive impairment?	Response 1&2: MMSE scale Response 3: Wechsler’s scale
Reason to the previous answer	Response 1&2: MMSE is an objective and concise measure of cognition. It is easy to administer and gives an objective assessment of cognitive impairment if present. Response 3: Wechsler’s scale measures adult and adolescent intelligence and hence cognition. Due to its unique scoring system it can even pinpoint learning disability.
Do you think optimum cognition has a link with brain health? If Yes Why?	Response 1: Cognition is just one aspect of brain function and if it’s optimum it results in a healthy brain. Response 2: The ability to perform all mental processes aptly like executive function, working memory and processing speed lead to execution of proper brain functions particularly cognition. Hence, a person with poor cognitive ability will definitely have poor brain health.

## DISCUSSION

There is a battery of tests to quantify various brain functions.^1^,^2^ Narrowing them down to just one test for each function is quite a technical task. This research narrowed down a range of clinical tests for measuring various brain functions to one best test for each brain function. These were Visual fields, Romberg’s test, Pure tone audiometry, TEIQue-SF, MMSE scale and Manual Muscle testing.

The criteria was selected on the basis of price per test for a patient, portability, level of skill needed for use, the time needed to perform, area Under the curve (AUC), diagnosing accuracy/validity/ reliability, usage in a clinical setup, and their link with brain health. These were selected keeping in mind not only the link with brain health and its functions, but also the feasibility of use in a clinical setup of a developing country.

The reason Visual Function (VF) was preferred over Visual Acuity (VA) despite both having the same overall score was that; AUC for visual fields falls between 0.9 and 1.0 17 and that for visual acuity is between 0.8 and 0.9.^16^ Similarly, accuracy for VA is 70-89%^17^,^18^ and that for VF falls between 90-100%.^19^ The link with brain function is higher for VF than for VA. It was for these three main reasons that the other qualities like portability, time and price were overlooked. Damage to the brain via insults like blast injuries can lead to visual defects like photophobia, difficulties in reading and convergence, accommodative insufficiency and complete or incomplete hemianopia.^36^ This can be due to damage to the afferent and efferent visual pathway as was well as the optic radiations and the occipital cortex. Sometimes axonal disruption in the corpus collosum and blood brain barrier damage can also lead to such defects.^37^

For hearing ability, BERA and PTA had almost the same score except for price per test per patient and the wide usage in a clinical setup for the latter. While the AUC for both fell between 0.6 and 0.8^20^, ^22^ and the accuracy reported was 90-100%.^21^,^23^ PTA was selected as the best test in the present research due to its wide scale use and cost effectiveness. Hearing loss is seen to cause gray matter atrophy particularly in areas related to Alzheimer’s disease.^38^ It also causes a decreases in thickness of auditory cortex and global cortical reorganization, resulting in conversion of auditory modalities/areas to other sensory modalities. Hearing loss also has an effect on other brain functions like cognition.^39^

For cognition, the most advocated scales were MMSE and Wechsler’s. MMSE took precedence in terms of price per patient (as it is freely available), level of skill needed to conduct it, the time needed per patient and accuracy, which was 70-89%.^27^ MMSE is most widely used and verified to screen cognitive impairment.^40^ Impaired cognition results in deterioration of brain health and even conditions like dementia and neurodegeneration. According to Duc et.al MMSE scores are related to brain cortical thinning and functional impairment.^40^

For motor testing, MMT was found to be the most appropriate test to be used in a clinical setup as it does not require any equipment and is widely practiced.^32^ Recurrent brain injury resulting in chronic brain damage, can lead to cortical and corpus callosal thinning and ventricular enlargement. It can also result in grip strength weakening and gait abnormalities.^41^ For emotional wellbeing TEIQue-SF was found to be the most useful scale due to it being free, having less time needed to fill, needing minimum expertise, and having high validity and reliability.^28^

### Strength of the study:

The strength of our study is the novel identification of the most efficient, cost-effective, accurate, and easy-to-use scales for measuring brain functions in the tertiary care clinical setup of a developing country. This is both novel and adds significance to the existing medical literature on the subject. Gauging of a tool used to measure brain function might help the primary health care worker sitting in the periphery of Pakistan to identify the underlying abnormality and in addition help them to suggest further investigations more specifically, it will also be cost-effective for all the stakeholders. In addition, the role of telemedicine might help to attain the standardization.

### Limitations:

The limitation of this research was that a limited number of experts were interviewed and the strength was that those interviews were augmented with an adequate literature review. This study will help clinicians of teaching hospitals as well as rural health setups to practice the most suitable tools for diagnosis. This will lead to a hassle-free clinical practice. The patients would be relieved from the inconvenience of going through a multitude of testing procedures.

## CONCLUSION

The best tools for gauging the above-mentioned brain functions are visual fields, PTA, MMSE, MMT, Romberg’s test and TEIQue-SF. These when measured, will provide the best representation of brain functions namely; visual function, hearing ability, cognition, motor function (muscle strength) and emotional well-being.

### Authors Contribution:

**UZ:** Data Collection, Analysis & Interpretation, Drafting the manuscript and Final Approval. Responsible for Accuracy and Integrity of the study

**SHH:** Conception & Design, Drafting the manuscript, Analysis & Interpretation and Final approval of the manuscript.

**SSR:** Data collection, analysis, and write up.
